# Drug use in pregnant women—a pilot study of the coherence between reported use of drugs and presence of drugs in plasma

**DOI:** 10.1007/s00228-017-2402-4

**Published:** 2017-12-20

**Authors:** Emelie Wolgast, Ann Josefsson, Martin Josefsson, Caroline Lilliecreutz, Margareta Reis

**Affiliations:** 1 Department of Obstetrics and Gynecology and Department of Clinical and Experimental Medicine, Linköping University, Linköping, Sweden; 2 Department of Forensic Genetics and Forensic Toxicology, National Board of Forensic Medicine, Linköping, Sweden; 3 Department of Physics, Chemistry and Biology, Linköping University, Linköping, Sweden; 4 Department of Clinical Pharmacology and Department of Medical and Health Sciences, Linköping University, Linköping, Sweden

**Keywords:** Drug screening, Time of flight mass spectrometry, Drug utilization, Interview, Pregnancy

## Abstract

**Purpose:**

In Sweden, information on drug use during pregnancy is obtained through an interview and recorded in a standardized medical record at every visit to the antenatal care clinic throughout the pregnancy. Antenatal, delivery, and neonatal records constitute the basis for the Swedish Medical Birth Register (MBR). The purpose of this exploratory study was to investigate the reliability of reported drug use by simultaneous screening for drug substances in the blood stream of the pregnant woman and thereby validate self-reported data in the MBR.

**Methods:**

Plasma samples from 200 women were obtained at gestational weeks 10–12 and 25 and screened for drugs by using ultra-high performance liquid chromatography with time of flight mass spectrometry (UHPLC-TOF-MS). The results from the analysis were then compared to medical records.

**Results:**

At the first sampling occasion, the drugs found by screening had been reported by 86% of the women and on the second sampling, 85.5%. Missed reported information was clearly associated with drugs for occasional use. The most common drugs in plasma taken in early and mid-pregnancy were meclizine and paracetamol. Two types of continuously used drugs, selective serotonin reuptake inhibitors and propranolol, were used. All women using them reported it and the drug screening revealed a 100% coherence.

**Conclusions:**

This study shows good coherence between reported drug intake and the drugs found in plasma samples, which in turn positively validates the MBR.

## Background

Pharmacotherapy during pregnancy is common [1, 2], not least when the woman has a chronic disease such as high blood pressure [3] or a psychiatric diagnosis [4]. However, adherence to the use of prescribed as well as over the counter (OTC) drugs is a well-known problem that may be particularly frequent among pregnant women owing to their fear of fetotoxic side effects [5] . When searching for the causality between negative effects such as a fetus malformation and/or neonatal morbidity due to drug exposure, noncompliance may bias studies in which the user information is based solely on prescription data and not including self-reported data [2] regardless of their respective biases.

The Swedish antenatal health care system reaches almost all pregnant women and is free of charge. Antenatal data, delivery, and neonatal records constitute the basis for the Swedish Medical Birth Register (MBR), a register containing information on practically all pregnancies in Sweden (1–2% missing) [6]. Since July 1, 1994, information on drug use during pregnancy has been forwarded to the MBR. It is obtained by an interview by a midwife and is recorded in a standardized medical record at the first antenatal care visit, as well as at all further visits throughout the pregnancy. Several hundred studies exploring possible fetotoxic effect are based on MBR (for a few examples, see [7–9]). In a study by Stephansson et al., a high agreement was found between self-reported drug use extracted from MBR and the Swedish Prescribed Drug Register (PDR) for drugs used for chronic conditions. However, it was low for occasional use [10]. Moreover, Källén et al. showed that self-reported data, in contrast to PDR data, appeared to give the most valid results at the first visit to the antenatal clinic compared to later reported information [11]. To monitor the presence of a substance in the blood stream at the time for self-reporting is an objective, however, time point-dependent method (i.e., it is depending on the half-life of the drug) to assess drug adherence.

New hyphenated analytical techniques such as ultra-high performance liquid chromatography with time of flight mass spectrometry (UHPLC-TOF-MS) allow broad targeted screening procedures in human specimens for therapeutic drugs as well as drugs of abuse. This analytical technique has recently been applied for investigations in clinical and forensic toxicology [12]. The aim of this study was to investigate the reliability of reported drug use at early pregnancy, gestational weeks (GW) 10–12, and mid-pregnancy GW 25, by simultaneous UHPLC-TOF-MS screening for drug substances in the plasma of pregnant women, to assess drug adherence and consequently the validity of MBR.

## Methods

In Sweden, healthy pregnant women are advised to attend the antenatal program consisting of seven to nine visits to a midwife, and, if needed, to make extra appointments with a physician and/or the midwife. The first visit generally takes place around GW 10–12 [6]. At the first visit, pregnant women are asked to report all drugs used from early pregnancy until the day of the interview. Thereafter, at each visit, the women are asked to report actual drugs used. All information is forwarded to MBR.

### Study population

In the biobank at the department of obstetrics and gynecology in Linköping, plasma samples from two occasions during pregnancy (GW 10–12 and GW 25) were collected for a number of years for study purposes. In 2013, plasma samples from the first 200 consecutively recruited women that year were withdrawn from the biobank. At both occasions the samples were obtained at the same day as the interview. The women were informed of the study objectives and gave their consent. In total, 400 plasma samples were analyzed.

### Plasma drug analysis

A targeted qualitative screening was performed by matching the MS results with an in-house database comprising more than 500 drugs and metabolites confirmed by the access of reference substance. For 240 of the included drugs, a minimum required performance level (MRPL) in blood was confirmed and validated by the analysis of standards of each compound in blood according to Roman et al. [13]. For some drugs, this concentration was at a high therapeutic level. In this work, though, all findings from the complete database that fulfilled the criteria for identification by TOF-MS were reported. The possibility of obtaining a positive finding through plasma screening versus blood was not expected to be adversely affected due to the less negative impact of the plasma matrix on the analysis.

Data were extracted by the “Find by Formula” algorithm and only those compounds whose retention time difference and mass errors were within the criteria set in the data analysis method were reported. A presumptive positive result included a match tolerance for mass error of ± 10 ppm, retention time deviation ± 0.15 min, and area of ≥ 30,000 counts. Identification was based on scoring of retention time, accurate mass measurement, and isotopic pattern. An overall score ≥ 75 out of 100 was considered as a positive result.

Finally, drugs registered in the antenatal medical records were compared with the findings from the plasma drug analysis. All relevant background data as well as drug use were prospectively recorded and extracted from the standardized antenatal medical records.

## Results

Characteristics of the study population are shown in Table 1.

**Table 1.** Characteristics of the study population (*n* = 200)

		*N* (%)
Socioeconomic groups	Unskilled workers	17 (8.5)
	Skilled workers	38 (19.0)
	Lower white-collar workers	55 (27.5)
	Middle/high white-collar workers and self-employed	55 (27.5)
	Students	23 (11.5)
	Unemployed	8 (4.0)
	Others	4 (2.0)
Body mass index	< 18.5	2 (1.0)
	18.5–24.9	106 (53.0)
	25–29.9	63 (31.5)
	> 30	29 (14.5)
Age	< 25	22 (11.0)
	25–29	74 (37.0)
	30–35	84 (42.0)
	36–	20 (10.0)
Marital status	Single parent	5 (2.5)
Parity	Primipara	66 (33.0)
	Multipara	134 (67.0)

All drugs considered, at the first sampling occasion, the drugs found by screening had been reported by 86% of the women. On the second sampling occasion, that number was 85.5%.

The absolute majority of drugs reported were for occasional use and OTC drugs. Only two types of continuously used drugs, selective serotonin reuptake inhibitors (SSRI; 5% of the women at the first visit and 5.5% in GW 25) and propranolol (*n* = 1), were used. A 100% coherence between bioanalytical screening and interview data was found for these.

In 17 of the 135 women (13%), who had reported no drug use at all in early pregnancy, drug substance(s) were found: 8 positive meclizine findings and 7 paracetamol findings, both OTC drugs and 4 promethazine, or promethazine in combination with ephedrine and caffeine findings.

In GW 25, 156 women reported that they did not use any drugs. However, drug substances were found in 21 of the women (13.5%): paracetamol in 16, meclizine in 5, omeprazole in 1, and phenylpropanolamine in 1 woman; all of these are OTC drugs, except for phenylpropanolamine. It applies at both sampling times that more than one substance could have been found in one single woman.

At the first antenatal care visit, 65 of 200 women (32.5%) reported the use of one or more drugs (vitamins and minerals supplements excluded) but the screening only detected drug substance/s in 45 women. At GW 25, 44 of 200 women (22%) reported the use of drug/s but drug screening detected substances in 46 women. Hence, 20 women at the first interview reported a drug use that not could be analytically verified and at GW 25, two women underreported their drug intake.

Self-reported drugs as well as all drugs found in UHPLC-TOF-MS screening are shown in Table 2. In some of the plasma samples, more than one drug was found. Drugs reported in medical records but, by technical analytical reasons, not found in screening were levothyroxine, inhalation drugs, mesalazine, antibiotics, lactulose, tinzaparin, acetylsalicylic acid, estradiol, progesterone, alginic acid, sterculia gum, ibuprofen, clemastine, and acyclovir.

**Table 2.** Drugs used in early and mid-pregnancy according to medical records (*n* = 200), drugs found by plasma drug screening (gestational weeks 10–12 and 25, *n* = 200), and deviations from reported drug intake

GW		Occasional use	Medical records (*n*)	Plasma screening (*n*)	Deviation
10 to 12	Antiemetic*	x	18	29	+ 11
	SSRI**		**10**	**10**	**0**
	Omeprazole	x	3	1	− 2
	Paracetamol	x	21	13	− 8
	Diazepam	x	1	1	0
	Cetirizine	x	2	1	− 1
	Sumatriptan	x	1	0	− 1
	Loperamide	x	1	0	− 1
	Ethylmorphine + cocillana	x	1	0	− 1
25	Antiemetic*	x	12	17	+ 5
	SSRI**		**11**	**11**	**0**
	Omeprazole	x	4	6	+ 2
	Paracetamol	x	4	20	+ 16
	Phenylpropanolamine	x	1	2	+ 1
	**Propranolol**		**1**	**1**	**0**
	Loperamide	x	0	1	− 1
	Sumatriptan	x	1	0	− 1

One woman can use more than one drug. Bold text indicates continuously used drugs

*Promethazine ± ephedrine and caffeine, meclizine, metoclopramide, ondansetron

A positive (+) deviation means that more women than had reported drug use had taken the drug at the time of the blood sampling

A negative (−) deviation means that fewer women had reported drug use than had taken the drug at the time of the blood sampling

**SSRI (selective serotonin reuptake inhibitor; citalopram, escitalopram, and sertraline), *GW* gestational week

The most common drug found in early pregnancy was meclizine, an over-the-counter antihistaminergic drug with antiemetic effects which 19 women used. The second most common found drug was paracetamol. In GW 25, the most common drug was paracetamol, which was found in 20 women, and the second most common was meclizine (*n* = 17) (Table 2).

## Discussion

In this descriptive study, we have found a good coherence between reported drug intake and presence of the drug in the pregnant women’s plasma; on the first sampling occasion, 86% and on the second, 85.5% had correctly reported drug use. Moreover, for drugs prescribed for continuous use, the coherence was 100%. As far as we know, this is the first study that has looked at the actual drug content in the plasma of pregnant women and then compared it to the reported drug use. A high correlation between self-reported data and drug screening strengthens the scientific outcome of the hundreds and counting of studies based on the MBR. This is a new way to indirectly improve the quality of internationally renowned register studies. Since it is an exploratory study and no power analysis could be done, data from 200 women were considered satisfactory in a first attempt to evaluate MBR.

The fact that more women reported use of drugs than was found with the plasma screening was expected. The standardized interview questions posed in early and mid-pregnancy differ. At the first antenatal visit, the woman is asked to report present use and to recall all drugs she has taken since she became pregnant, i.e., the drug content in blood is time point-dependent and historic use is not detected. At the second blood sampling in GW 25, the woman is asked which drugs she is taking at the present date. Many of the drugs reported are not taken on a regular basis, e.g., paracetamol or antiemetic drugs. Furthermore, depending on the half-life of a drug, it can be traced during a shorter or longer period of time. For example, paracetamol has a half-life of 2 h and meclizine has a half-life of 6 h. By comparison, sertraline has a half-life of 26 h and citalopram, 36 h. There is no record of the timespan between intake and blood sampling.

The most common drugs in the plasma samples taken in early and mid-pregnancy were meclizine and paracetamol. The same two drugs were also found in 13% of the women who reported no use of drugs. A possible explanation might be that neither of these two drugs is considered as “actual drugs” since they are over the counter drugs. Another possible explanation is recall bias, which has been shown to be more common for drugs used irregularly [14].

A weakness, as well as a strength, of the study design is that data was based on the reported drug use in the antenatal records and not from the actual MBR. The Swedish MBR is a unique register based on standardized antenatal medical records transferred from the antenatal care system [6]. However, as all registers, it has weaknesses. For example, data obtained in the antenatal medical records could be misinterpreted or misspelled by the individual midwife. In the light, though, of more than 100,000 pregnancy records transferred to MBR per year the overall risk for population errors are to be considered marginal.

A study weakness is that not all drugs, for analytical technical reasons, are detectable in the applied UHPLC-TOF-MS screening. However, the data in the present study supported the findings by Stephansson et al. [10] of a high agreement between self-reported MBR drug data and PDR for chronic conditions but low agreement for occasional use. For example, all the women who had SSRI found by screening also had reported a use of an SSRI.

## Conclusions

There was a good coherence between reported drug intake and the drugs found in the plasma samples which in turn positively validated the MBR. The clinical implication of this study is that the pregnant women’s self-reported drug use is reliable. The results from this relatively small population call for future larger studies applying the same methods, validating self-reported data with actual biochemical findings.
